# Supplementary material for: Asynchronous magnetic resonance elastography: Shear wave speed reconstruction using noise correlation of incoherent waves
Source: Magn Reson Med. 2022 Oct 27;89(3):990–1001. doi: 10.1002/mrm.29502 (PMC9792433; doi:10.1002/mrm.29502)
Supplement: Supplementary file 1 — DATA S1 MATLAB code used to generate the numerical simulations of Figure 1 [file MRM-89-990-s002.zip › k-Wave/helpfiles/h5compare.html]

h5compare :: Functions (k-Wave)


# h5compare

Compare the contents of two HDF5 files.

## Syntax

```
h5compare(filename1, filename2)
```

## Description

`h5compare` compares the datasets and attributes within two HDF5 files. Fields that are present in one file but not the other are reported as missing. For fields that exist in both files but have different values, the L\_inf error is reported. All outputs are printed to the command line.

## Inputs

|  |  |
| --- | --- |
| `filename1` | filename of the first HDF5 file |
| `filename2` | filename of the second HDF5 file |

## See Also

`h5write`, `writeMatrix`
